# Obesity Index That Better Predict Metabolic Syndrome: Body Mass Index, Waist Circumference, Waist Hip Ratio, or Waist Height Ratio

**DOI:** 10.1155/2013/269038

**Published:** 2013-08-13

**Authors:** Abdulbari Bener, Mohammad T. Yousafzai, Sarah Darwish, Abdulla O. A. A. Al-Hamaq, Eman A. Nasralla, Mohammad Abdul-Ghani

**Affiliations:** ^1^Department of Medical Statistics & Epidemiology, Hamad Medical Corporation, P.O. Box 3050, Doha, Qatar; ^2^Department of Public Health, Weill Cornell Medical College, P.O. Box 3050, Doha, Qatar; ^3^Department Evidence for Population Health Unit, School of Epidemiology and Health Sciences, The University of Manchester, Manchester, UK; ^4^Department of Endocrinology, Hamad General Hospital, Hamad Medical Corporation, P.O. Box 3050, Doha, Qatar; ^5^Qatar Diabetic Association and Qatar Foundation, P.O. Box 752, Doha, Qatar; ^6^Qatar Foundation Research Division, P.O. Box 5825, Doha, Qatar; ^7^Division of Diabetes, University of Texas Health Science Center at San Antonio, 7703 Floyd Curl Drive, San Antonio, TX 78229, USA

## Abstract

*Aim*. The aim was to compare body mass index (BMI), waist circumference (WC), waist hip ratio (WHR), and waist height ratio (WHtR) to identify the best predictor of metabolic syndrome (MetS) among Qatari adult population. *Methods*. A cross-sectional survey from April 2011 to December 2012. Data was collected from 1552 participants followed by blood sampling. MetS was defined according to Third Adult Treatment Panel (ATPIII) and International Diabetes Federation (IDF). Receiver operating characteristics (ROC) curve analysis was performed. *Results*. Among men, WC followed by WHR and WHtR yielded the highest area under the curve (AUC) (0.78; 95% CI 0.74–0.82 and 0.75; 95% CI 0.71–0.79, resp.). Among women, WC followed by WHtR yielded the highest AUC (0.81; 95% CI 0.78–0.85 & 0.79; 95% CI 0.76–0.83, resp.). Among men, WC at a cut-off 99.5 cm resulted in the highest Youden index with sensitivity 81.6% and 63.9% specificity. Among women, WC at a cut-off 91 cm resulted in the highest Youden index with the corresponding sensitivity and specificity of 86.5% and 64.7%, respectively. BMI had the lowest sensitivity and specificity in both genders. *Conclusion*. WC at cut-off 99.5 cm in men and 91 cm in women was the best predictor of MetS in Qatar.

## 1. Introduction

Metabolic syndrome (MetS) is known to be a cluster of interrelated risk factors of metabolic origin such as elevated blood pressures, glucose metabolism disturbances, dyslipidemia, and obesity [1], which are linked to the development of atherosclerotic cardiovascular diseases and type 2 diabetes mellitus (T2DM) [1–4]. Out of all these risk factors, obesity or body fat seems to be the predominant underlying risk factor not only in the development of MetS but also other cardiovascular risk factors [5, 6]. 

It has been widely considered that central obesity as a marker of body fat can be conveniently and cost effectively estimated by measuring body mass index (BMI) and waist circumference (WC) that in turn might effectively predict the risk of MetS [7, 8]. Recently some other indices of abdominal obesity such as waist hip ratio (WHR) and waist height ratio (WHtR) have also been reported to be better discriminators of cardiovascular and metabolic risk factors than BMI and/or WC. However, studies from different countries and ethnicities have different conclusions regarding the superiority of one or the other obesity index and related cut-off points to diagnose obesity and hence MetS [9–11]. Researchers believe that ethnic and racial variation among population from different regions might need different cut-off points and/or use of different anthropometric measurement to diagnose obesity and MetS [10, 11]. To the best of our knowledge no previous studies in Qatar or neighboring Arab countries have investigated the best indicator for central obesity and related locally appropriate cut-off point for the prediction and diagnosis of MetS among Arab population. Therefore, we conducted this study with the aim to evaluate different obesity indices (BMI, WC, WHR, and WHtR) to identify the one that better predicts metabolic syndrome (MetS) and its related sex-specific optimal cut-off points among Qatari population. 

## 2. Subjects and Methods

We conducted a cross-sectional study among the adult Qatari population above 20 years of age over a period from April 2011 to December 2012. The study protocol was approved by the Ethics committee of Hamad Medical Corporation. Each participant was provided with brief information about the study and was assured of strict confidentiality. Only participants who agreed to participate and gave written consent were included in the study.

### 2.1. Sampling Procedure

We developed a multistage stratified cluster sampling design using the administrative divisions of the primary health centres (PHC) in Qatar that had approximately equal population coverage. In order to secure a representative sample of the study population, sampling was stratified with a view to obtain proportional representation from urban and semiurban areas. The sample size was determined based on a priori assumption for the prevalence of MetS in Qatar and Eastern Mediterranean region to be 17–20%; with the 99% confidence interval and 2% bound on error of estimation, a minimum sample size of 2,182 would be required for this study. Out of total 22 PHCs available, 13 were selected at random. Of these 10 were located in urban and 3 in semiurban areas of Qatar. Lastly, subjects were selected systematically 1-in-2 sampling procedure. During the study period, 2,182 subjects were approached, of whom 1,552 (71%) consented to participate.

### 2.2. Questionnaire

We used a well designed and pilot tested questionnaire to collect the data. The questionnaire was tested among 100 subjects as a pilot study before the initiation of the main survey. We made necessary corrections and modifications in the questionnaire based on the findings from the pilot study. The first part of the questionnaire included information about sociodemographic and anthropometric characteristics including age, sex, marital status, education level, occupation, height, weight, and waist and hip circumference. The second part consisted of life style habits such as physical activity, fast food consumption and smoking habits. Last part of the questionnaire comprised of information about systolic and diastolic blood pressures, serum triglyceride, total cholesterol, high-density lipoprotein (HDL) cholesterol, low density lipoprotein (LDL) cholesterol, Hemoglobin A1c (HbA1c), and fasting plasma glucose levels (FPG). 

### 2.3. Diagnostic Criteria

We used two different international criteria as given below to diagnose MetS among the participants.

1. National Cholesterol Education Program: Third Adult Treatment Panel (ATP III) [1].
2. According to ATPIII, presence of at least three of these risk factors diagnose the MetS: (a) FPG ≥ 100 mg/dL (5.6 mmol/L); (b) blood Pressure ≥ 130/85 mmHg; (c) triglyceride ≥ 150 mg/dL (1.7 mmol/L); (d) HDL Cholesterol: Men < 40 mg/dL (1.03 mmol/L); women < 50 mg/dL (1.29 mmol/L); (e) men with waist circumference >102 cm and women with waist circumference >88 cm.
3. International Diabetes Federation (IDF) [12].
4. According to IDF, a participant has the MetS if she/he has a waist circumference (≥94 cm in men and ≥80 cm in women) plus any two of these risk factors: (a) FPG ≥ 100 mg/dL (5.6 mmol/L) or previously diagnosed impaired fasting glucose (b) blood pressure ≥ 130/85 mmHg or treatment for hypertension; (c) Triglyceride ≥ 150 mg/dL (1.7 mmol/L); (d) HDL Cholesterol: Men < 40 mg/dL (1.03 mmol/L); Women < 50 mg/dL (1.29 mmol/L) or treatment for low HDL.

### 2.4. Anthropometric and Blood Pressure Measurements

Physical examination and measurements were performed by a trained nurse. Height was measured in centimetres (cm) using a height scale (SECA, Germany) while the subject was standing bare feet and with normal straight posture. Male subjects were requested to remove their head cover (*Igaal and Guttra*). Weight was measured in kilograms using a weight scale (SECA, Germany). The subjects were asked to remove any objects from their pockets and to stand on the weight scale bare feet with light clothing. BMI was calculated as the ratio of weight (kilogram) to the square of height (meters). Obesity and overweight were classified according to WHO criteria [13]. A person was considered obese if the BMI value was ≥30 kg/m^2^, overweight if BMI ≥25 kg/m^2^ and <30 kg/m^2^. 

Waist circumference was measured in centimetres without compression of the soft tissue at midway level between lower rib margin and iliac crest using nonstretchable measuring tape. The hip circumference was also measured in centimetres using the same measuring tape at its widest portion of the buttocks, with the tape parallel to the floor. Both measurements were taken while the subject was standing with feet closed together, arms at the side, body weight evenly distributed, and wearing little clothing. Also, the measurements were taken at the end of a normal expiration. Waist to Hip ratio (WHR) was calculated by taking the waist circumference (cm) and dividing by the hip circumference (cm) while on the other hand Waist to Height ratio (WHtR) was calculated by taking waist circumference (cm) and dividing by height (cm). 

Two readings of the systolic (SBP) and diastolic (DBP) blood pressure were taken from the subject's left arm while seated and his/her arm at heart level, using a standard zero mercury sphygmomanometer after at least 10–15 minutes of rest. Then the average of the two readings was obtained. 

### 2.5. Laboratory Measurements

A blood sample of 10 mL was collected through venipuncture from each participant after fasting for 10 hours, into vacutainer tubes containing EDTA. The samples were kept at room temperature and transported within 2 hrs to a central certified laboratory at Hamad General Hospital, HMC, Doha, Qatar. Plasma glucose, total cholesterol, triglyceride, HDL-cholesterol, and LDL-cholesterol were measured by an autoanalyser (Hitachi 747 autoanalyzer, Japan). 

### 2.6. Assessment of Lifestyle Factors

Information on cigarette and sheesha smoking was obtained separately by asking questions regarding the smoking status (never smoke, past smoker and current smoker); if smoker then further questions were asked regarding number of years smoked and number of cigarettes smoked per day. Information on physical activity/exercise was also obtained subjectively by asking question regarding any activity causing light perspiration or slight to moderate increase in breathing or heart rate for at least 30 minutes which is performed regularly (yes/no). Information about fast food consumption was measured through a single binary response question “do you eat fast foods? Yes/No.” 

### 2.7. Data Analysis

Data were analyzed using the Statistical Package for Social Sciences version 20 (SPSS Inc., Chicago, IL, USA) software. Continuous variables were tested for normality using histograms and Kolmogorov-Smirnov test. Continuous variables were expressed as mean with standard deviation and categorical variables were expressed as frequency with percentage. Comparison of sociodemographic variables, lifestyle habits, and anthropometric and biochemical measurements between subjects with and without MetS was made using Pearson chi square for categorical variables and independent samples Student's *t*-test for continuous scale variables. The receiver operating characteristic (ROC) curve was generated to obtain the values of area under the curve (AUC) with 95% CI, and also sensitivity and specificity for each obesity index as a predictor of MetS. To determine the locally appropriate sex-specific cut-off point for each obesity index, the Youden index (sensitivity + specificity − 1) was calculated and the corresponding cut-off value for the highest Youden index was considered as the optimal cut-off value. 

In addition, multivariable logistic regression analysis was conducted for total population, males and females separately to identify the strength of association of different obesity indices (adjusting for age, education, smoking status, family history of hypertension and diabetes) and MetS.

## 3. Results

### 3.1. Baseline Characteristics

Overall, the prevalence of MetS was 26.2% according to ATPIII and 36.9% according to IDF.  Table 1 shows comparison of sociodemographic and lifestyle characteristics between participants with and without MetS in Qatar. Participants with MetS were older, predominantly female and were either retired/not working or housewives as compared to those without MetS using ATPIII criteria (45.93 ± 11.1 versus 41.50 ± 11.30; *P* < 0.001, 57% versus 49%; *P* = 0.005, and 47% versus 39%; *P* = 0.024, resp.); however, no such difference was observed between the groups based on IDF criteria (*P* > 0.05). Level of education, consumption of fast food, and physical activity were significantly different between groups with and without MetS using both the diagnostic criteria. 

Almost half of the participants with MetS were obese (BMI ≥ 30) as compared to slightly higher than one third of the metabolically healthy obese individuals (58.6% versus 37.1% using ATPIII criteria and 49.1% versus 39% using IDF criteria; *P* < 0.001, resp.). Average WC, WHR, WHtR, BMI, FPG, triglycerides, SBP, and DBP were significantly higher among the participants with MetS as compared to those without MetS irrespective of the diagnostic criteria. In contrast, average HDL cholesterol was significantly lower in MetS than without MetS (1.32 ± 0.27 versus 1.42 ± 0.34; *P* = 0.032 using ATPIII criteria and 1.36 ± 0.30 versus 1.42 ± 0.34; *P* = 0.001 using IDF criteria) (Table 2).

### 3.2. Obesity Indices and Metabolic Syndrome Using ROC Curves

**Figure 1:**
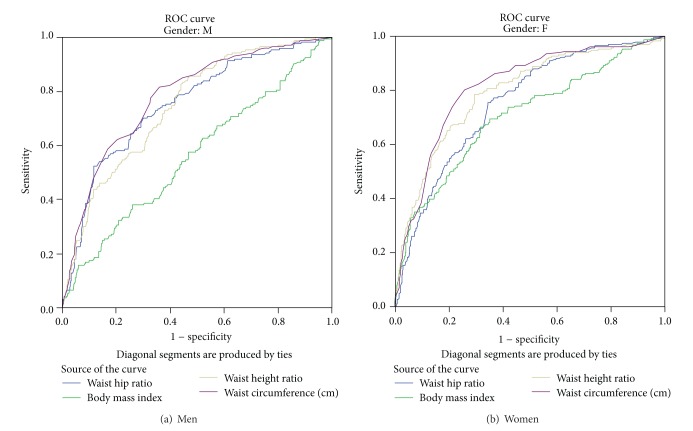
Receiver operating characteristics curve (ROC).

Table 3 and Figure 1 show gender specific area under ROC curve and optimal cut-off points with corresponding validity parameters for different obesity indices in predicting MetS. Among men, WC followed by both WHR and WHtR yielded the highest AUC (0.78; 95% CI 0.74–0.82 and 0.75; 95% CI 0.71–0.79, resp.). Unlike men, among women WC followed by WHtR yielded the highest area under the curve (0.81; 95% CI 0.78–0.85 and 0.79; 95% CI 0.76–0.83). BMI produced the lowest AUC in both men and women (0.56; 95% CI 0.51–0.61 and 0.70; 95% CI 0.66–0.74, resp.). 

Among men, WC at a cut-off value of 99.5 cm resulted in the highest Youden index with corresponding sensitivity of 81.6% and 63.9% specificity. At a traditional cut-off value of 102 cm of WC for men, the sensitivity dropped to 75.9%, and specificity slightly raised to 67.3%. Similarly among women, WC at a cut-off point of 91 cm resulted in highest Youden index with the corresponding sensitivity and specificity of 86.5% and 64.7%, respectively. At a traditional cut-off point of 88 cm WC among women, the sensitivity steeply increased to 94.4%, but this happened at the expense of significant drop in the specificity from 64.7% to 53.2%. Among both men and women, the BMI at a cut-off value of 28 kg/m^2^ and the traditional cut-off value of 30 kg/m^2^ were found to be having the lowest Youden index and corresponding sensitivity and specificity. WC at a cut-off point of 99.5 cm among men and 91 cm among women happened to be the best predictor of metabolic syndrome in Qatari population.

**Figure 2:**
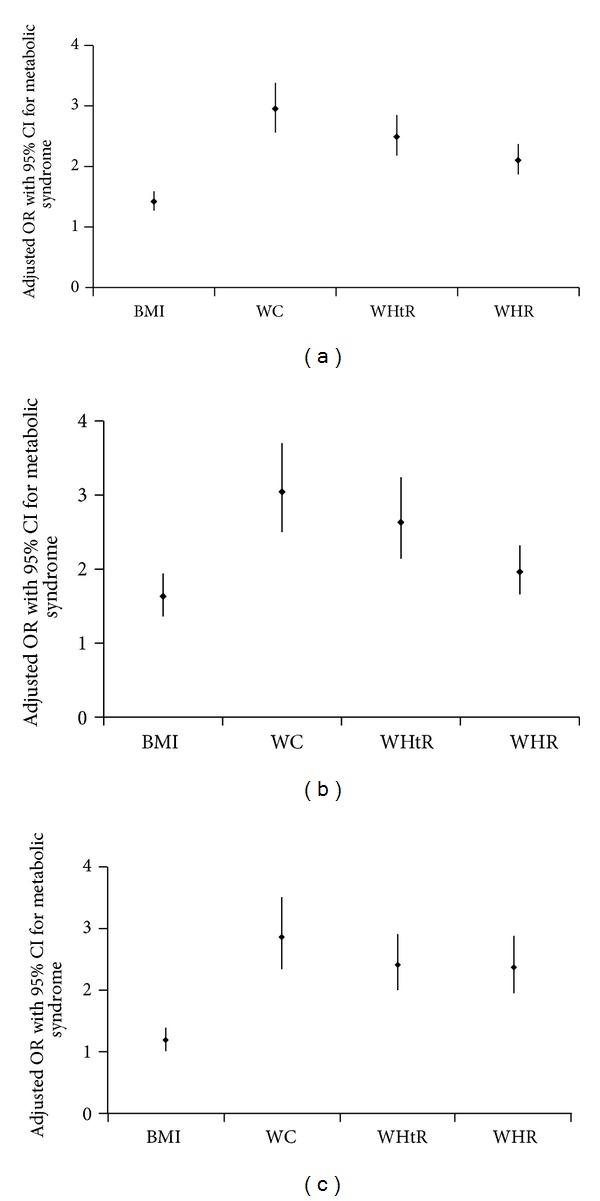
Adjusted odds ratios (adj. OR) for metabolic syndrome for one quartile increase in anthropometric variables in individuals. All the models are adjusted for age, education, smoking status, and family history of hypertension and diabetes. (a) Model for the general population, (b) model for females population, and (c) model for male population. BMI = Body Mass Index, WC = Waist Circumference, WHtR = Waist Height Ratio, and WHR = Waist Hip Ratio.

Figure 2 shows that adjusted odds ratios (OR) for metabolic syndrome for one quartile increase in anthropometric variables in Qatari population. All the models are adjusted for age, education, smoking status, and family history of hypertension and diabetes: (a) model for the general population, (b) model for female population, and (c) model for male population. The figure shows that the adjusted odds ratio of MetS is the highest for one quartile increase in the WC as compared to all the other indices of obesity irrespective of gender. 

## 4. Discussion

 In this cross-sectional survey of Qatari nationals aged 20 years and above, we found that the overall prevalence of MetS was 26% according to ATPIII and 37% according to IDF criteria which was consistent with the previous study conducted among Qatari adult population [2]. In addition, as per the main aim of this study we found that WC was a better predictor of MetS as compared to other obesity indices such as BMI, WHR, and WHtR in both men and women. The optimal cut-off values of WC to predict MetS were 99.5 cm and 91 cm in men and women, respectively. Those of WHR, WHtR, and BMI were 0.90 and 0.88, 0.58 and 0.63, 28 kg/m^2^ and 28.4 kg/m^2^ in men and women, respectively.

 Which measure of obesity should be used for predicting MetS is widely debated. A recent cross-sectional survey among adult Iranian population found that WC was superior to BMI and WHR in discriminating MetS among healthy individuals [11]. Similarly, another cross-sectional survey among Whites and African American adult population in US reported WC to be the most powerful tool in predicting MetS and BMI was inferior to WC among men in general but not women [6]. However, a cross-sectional survey among Chinese adult population reported WC, WHR, and BMI as equally useful indicators to discriminate between those with and without MetS [14]. Among Cohort studies, the San Antonio Heart Study reported both BMI and WC as having equal power in predicting development of MetS in non-Hispanic Whites and Mexican Americans [15]. While on the other hand, a follow-up study among Korean adults found WHR as better predictor of multiple metabolic risk factors than WC, WHtR, and BMI [10]. On the contrary, an INTERHEART study among a large cohort of primary care patients reported WHtR as a better predictor of metabolic risk factors except hypertension as compared to other obesity indices, while interestingly BMI was found to be the better predictor of hypertension alone as compared to other obesity measurements [16]. These ethnic variations in results suggest that predictive power of each obesity index differ by ethnic group and therefore we believe that the discrete decision to select particular obesity index for diagnosis of MetS should be specific to each ethnic population. Based on this study, we found WC as a better predictor of MetS in both Qatari men and women. Our results are robust after adjusting for known confounders such as age, education, smoking, and family history of diabetes and hypertension using multivariable logistic regression. 

 Irrespective of all these ethnic variations regarding appropriateness of obesity indices, studies have shown that BMI poorly discriminates between excess adipose tissue and high lean muscle mass and that it does not account for body fat distribution [17, 18]. On the other hand, WC is reported to be better correlated with abdominal fat and strongly associated with cardiovascular risk factors than BMI [19–21]. Therefore, WC is most commonly recommended to assess cardiovascular risk factors and is widely used in the definition of MetS [1, 12, 22]. Nevertheless, some researchers reported that measuring WC alone as a surrogate for abdominal fat distribution might overestimate the risk of MetS in tall subjects and underestimate in short subjects [23]. Therefore, they prefer WC adjusted for height as WHtR a better surrogate for measuring abdominal obesity [24, 25]. However, follow-up studies in Denmark and Japan did not support the importance of WC adjusted for height (WHtR) as a measure of adiposity in both men and women to identify metabolic risk factors [26, 27]. 

 In this study we found that WC at a cut-off value of 99.5 cm for men and 91 cm for women has the highest sensitivity and specificity to predict the development of MetS. When we applied the WC cut-off value of 102 cm for men and 88 cm for women as recommended by ATPIII criteria [1], the sensitivity to discriminate between those with and without MetS dropped from 81.6% to 75.9% in men and the specificity dropped from 64.7% to 53.2% among women. A study among Iran adult population reported WC cut-off point of 90.3 cm among women which is similar to our findings, but their reported cut-off point for men was 90 cm which is lower than our cut-off for men [11]. Another study from Basra city in Iraq also reported slightly different WC cut-off value of 97 cm for men and 99 cm for women to diagnose MetS using the IDF criteria [28]. These minor differences in the cut-off values might be attributed to ethnic variations and using different criteria for diagnosing MetS. Nevertheless, similar to our finding all these studies show that cut-off points lower than currently recommended by ATPIII criteria for WC are needed for men while higher cut-off points are suggested for women to identify MetS among adult population in the region.

 Our results also show that, after the WC, WHR at a cut-off point of 0.90 for men and 0.88 for women yields the highest sensitivity and specificity to differentiate MetS. This finding is consistent with previous studies from Oman, Korea, and Iran [8, 10, 11]. We found BMI to be the poorest predictor of MetS as compared to other obesity indices. Yet, at a slightly lower cut-off value of 28 kg/m^2^ than the recommended cut-off point by WHO [13] for both men and women it produced better sensitivity and specificity to predict the risk of MetS. Previous studies in Iran, Oman, and Korea also suggest using the lower cut-off points for BMI to better predict the development of MetS [11]. 

Our study has two important limitations. Firstly, the cross-sectional design of this study has inherent limitation of temporality and hence inability to draw causal inferences. Secondly, we could not adjust our sample size calculation for the potential nonresponse bias. However, despite these limitations we were able to recruit a large number of representative samples with a response rate of 71% from both rural and urban areas of Qatar and our results are generalizable to the entire Qatari adult population. 

## 5. Conclusion

Waist circumference at a cut-off point of 99.5 cm among men and 91 cm among women happened to be the best predictor of metabolic syndrome in Qatari population. Using the traditional cut-off values of 102 cm for men and 88 cm in women as recommended by ATPIII criteria for the Arab region might result in underestimation of MetS among men and overestimation among women. Looking at the discrepancies we recommend that WHO, IDF, and ATPIII criteria for obesity might not be appropriate for predicting the risk of MetS among Qatari adult population.

## Figures and Tables

**Table 1:** Demographic and lifestyle habits of the study sample in Qatar (*N* = 1552).

Variables	Total *N* = 1552	ATPIII		*P* value	IDF		*P* value
		MetS(+) *n* = 406	MetS(−) *n* = 1146		MetS(+) *n* = 572	MetS(−) *n* = 980	
	*N* (%)	*n* (%)	*n* (%)		*n* (%)	*n* (%)	
Age (mean ± SD)	42.66 ± 11.1	45.93 ± 10.03	41.50 ± 11.30	<0.001	42.89 ± 10.73	42.52 ± 11.41	0.534
Age groups (years)							
<35	411 (26.5)	359 (31.3)	52 (12.8)	<0.001	137 (24.0)	274 (28.0)	0.239
35–44.9	461 (29.7)	333 (29.1)	128 (31.5)		177 (30.9)	284 (29.0)	
45–54.9	455 (29.3)	314 (27.4)	141 (34.7)		182 (31.8)	273 (27.9)	
55–64.9	175 (11.3)	102 (8.9)	73 (18.0)		59 (10.3)	116 (11.8)	
65 and above	50 (3.2)	38 (3.3)	12 (3.0)		17 (3.0)	33 (3.4)	
Male gender	758 (48.8)	174 (42.9)	584 (51.0)	0.005	274 (47.9)	484 (49.4)	0.572
Education level							
<Secondary	863 (55.6)	259 (63.8)	604 (52.7)	<0.001	358 (62.6)	505 (51.5)	0.001
≥Secondary	689 (44.4)	147 (36.2)	542 (47.3)		214 (37.4)	475 (48.5)	
Occupation							
Retired/not working/HW*	643 (41.4)	191 (47.0)	452 (39.4)	0.024	241 (42.1)	402 (41.0)	0.911
Professional	756 (48.7)	176 (43.3)	580 (50.6)		275 (48.1)	481 (49.1)	
Manual worker	153 (9.9)	39 (9.6)	114 (9.9)		56 (9.8)	97 (9.9)	
Type of residence							
Shabia	517 (33.1)	121 (29.8)	396 (34.6)	0.130	178 (31.1)	339 (34.6)	0.346
Villa	909 (58.8)	255 (62.8)	654 (57.1)		344 (60.1)	565 (57.7)	
Apartment	126 (8.1)	30 (7.4)	96 (8.4)		50 (8.7)	76 (7.8)	
Marital status							
Married	934 (60.2)	241 (59.4)	693 (60.5)	0.694	360 (62.9)	574 (58.6)	0.090
Single	618 (39.8)	165 (40.6)	453 (39.5)		212 (37.1)	406 (41.4)	
Fast food consumption	518 (33.4)	164 (40.4)	354 (30.9)	<0.001	217 (37.9)	301 (30.7)	0.004
Physical activity	422 (27.2)	86 (21.2)	336 (29.3)	0.002	134 (23.4)	288 (29.4)	0.011
Smoking status							
Never smoke	1246 (80.3)	332 (81.8)	914 (79.8)	0.337	459 (80.2)	787 (80.3)	0.145
Past smoker	147 (9.5)	31 (7.6)	116 (10.1)		46 (8.0)	101 (10.3)	
Current smoker	159 (10.2)	43 (10.6)	116 (10.1)		67 (11.7)	92 (9.4)	
Avg. number of years smoked	13.25 ± 9.53	13.28 ± 11.60	13.24 ± 8.86	0.984	12.99 ± 8.70	13.71 ± 10.89	0.624
No. of cigarettes smoke/day (mean ± SD)	19.63 ± 13.01	22.44 ± 12.71	18.81 ± 13.02	0.099	18.93 ± 13.38	20.03 ± 12.83	0.567
Sheesha smoking	332 (21.4)	83 (20.4)	249 (21.7)	0.588	135 (23.6)	197 (20.1)	0.105

*HW: housewife.

**Table 2:** Anthropometric measurements and clinical parameters of the study sample in Qatar (*N* = 1,552).

Variables	Total *N* = 1,552	ATPIII		*P* value	IDF		*P* value
		MetS(+) *n* = 406	MetS(−) *n* = 1146		MetS(+) *n* = 572	MetS(−) *n* = 980	
	*N* (%)	*n* (%)	*n* (%)		*n* (%)	*n* (%)	
Waist circumference (cm)	97.13 ± 12.40	106.04 ± 10.17	93.97 ± 11.56	<0.001	98.87 ± 13.23	96.11 ± 11.78	<0.001
Hip circumference (cm)	110.02 ± 9.87	113.58 ± 10.27	108.75 ± 9.42	<0.001	110.62 ± 10.85	109.67 ± 9.24	0.068
Height (cm)	162.84 ± 9.65	162.33 ± 10.03	163.02 ± 9.51	0.215	162.84 ± 9.65	162.37 ± 9.57	0.139
Weight (Kg)	77.51 ± 15.97	83.35 ± 16.92	75.44 ± 15.09	<0.001	78.92 ± 18.10	76.69 ± 14.52	0.008
Waist hip ratio (WHR)	0.88 ± 0.09	0.94 ± 0.08	0.86 ± 0.08	<0.001	0.89 ± 0.09	0.88 ± 0.09	<0.001
Waist height ratio	0.60 ± 0.08	0.66 ± 0.07	0.58 ± 0.08	<0.001	0.61 ± 0.09	0.59 ± 0.08	<0.001
Body mass index (Kg/m^2^)	29.32 ± 6.10	31.87 ± 7.11	28.42 ± 5.41	<0.001	30.10 ± 7.31	28.86 ± 5.21	<0.001
Body mass index: *n* (%)							
<25	377 (24.3)	72 (17.7)	305 (26.6)	<0.001	164 (28.7)	213 (21.7)	<0.001
25–29.9	512 (33.0)	96 (23.6)	416 (36.3)		127 (22.2)	385 (39.3)	
≥30	663 (42.7)	238 (58.6)	425 (37.1)		281 (49.1)	382 (39.0)	
Fasting glucose (mmol/L)	6.26 ± 2.54	8.31 ± 3.43	5.54 ± 1.62	<0.001	6.97 ± 3.04	5.85 ± 1.30	<0.001
Haemoglobin A1c (%)	6.11 ± 1.59	7.36 ± 1.93	5.67 ± 1.18	<0.001	6.58 ± 1.92	5.84 ± 1.30	<0.001
Total cholesterol (mmol/L)	4.86 ± 0.83	5.00 ± 0.83	4.81 ± 0.82	<0.001	4.93 ± 0.84	4.81 ± 0.82	0.006
HDL cholesterol (mmol/L)	1.40 ± 0.33	1.32 ± 0.27	1.42 ± 0.34	<0.001	1.36 ± 0.30	1.42 ± 0.34	0.001
LDL cholesterol (mmol/L)	2.81 ± 0.70	2.88 ± 0.68	2.79 ± 0.70	0.032	2.83 ± 0.71	2.80 ± 0.69	0.552
Triglycerides (mmol/L)	1.41 ± 0.81	1.63 ± 0.82	1.34 ± 0.79	<0.001	1.48 ± 0.76	1.37 ± 0.83	0.010
Systolic blood pressure (mmHg)	127.36 ± 16.00	131.57 ± 15.41	125.87 ± 15.95	<0.001	128.58 ± 16.23	126.65 ± 15.84	0.023
Diastolic blood pressure (mmHg)	79.23 ± 9.75	82.16 ± 9.42	78.19 ± 9.65	<0.001	80.32 ± 10.18	78.59 ± 9.43	<0.001

**Table 3:** Area under the ROC curve, optimal cut-off points, and validity parameters of different obesity indices in predicting MetS (*N* = 1,552).

	AUC (95% CI)	Cut-off value	Sensitivity	Specificity	Youden index
Men					
Body mass index (BMI)	0.56 (0.51–0.61)	28 kg/m^2^	58.0%	52.9%	0.109
		30 kg/m^2^	38.5%	66.7%	0.052
Waist circumference (WC)	0.78 (0.74–0.82)	99.5 cm	81.6%	63.9%	0.455
		102 cm	75.9%	67.3%	0.432
Waist height ratio (WHtR)	0.74 (0.71–0.79)	0.58	75.1%	64.8%	0.399
		0.50	96.6%	24.5%	0.211
Waist hip ratio (WHR)	0.75 (0.71–0.79)	0.90	70.1%	69.9%	0.400
Women					
Body mass index (BMI)	0.70 (0.66–0.74)	28.4 kg/m^2^	73.7%	64.8%	0.385
		30 kg/m^2^	66.4%	67.1%	0.335
Waist circumference (WC)	0.81 (0.78–0.85)	91.0 cm	86.5%	64.7%	0.512
		88 cm	94.4%	53.2%	0.476
Waist height ratio (WHtR)	0.79 (0.76–0.83)	0.63	77.6%	71.5%	0.491
		0.50	96.1%	20.1%	0.162
Waist hip ratio (WHR)	0.75 (0.72–0.79)	0.88	75.4%	71.5%	0.469

AUC: area under the curve, ROC: receiver operating characteristics, CI: confidence interval.
